# Prophylactic Administration of Engineered *Bacillus subtilis* Expressing Mucosal Repair Factors Alleviates Pullorum Disease in Chicks

**DOI:** 10.3390/microorganisms14081606

**Published:** 2026-07-23

**Authors:** Fei Teng, Yingying Ma, Xinrui Li, Rongyan Li, Yang Yang, Yue Yan, Hongzhe Zhao, Guiwei Li, Yanping Jiang, Jiaxuan Li, Wen Cui, Xinyuan Qiao

**Affiliations:** 1 Heilongjiang Key Laboratory for Animal Disease Control and Pharmaceutical Development, Department of Preventive Veterinary, College of Veterinary, Northeast Agricultural University, 600 Changjiang Road, Harbin 150030, China; teng1085579571@163.com (F.T.); myy930823@163.com (Y.M.);; 2 Institute of Rural Revitalization Science and Technology, Heilongjiang Academy of Agricultural Sciences, Harbin 150030, China

**Keywords:** engineered probiotics, *Bacillus subtilis*, *Salmonella pullorum*, poultry health, mucosal protection, antibiotic alternatives

## Abstract

*Salmonella pullorum* (*S. pullorum*) remains a major enteric pathogen in young chicks, causing high mortality and severe economic losses in poultry production. This study evaluated a prophylactic strategy using engineered *Bacillus subtilis* (*B. subtilis*) expressing gallus trefoil factor 2 (gTFF2) and epidermal growth factor (gEGF) to protect chicks against *S. pullorum* infection. In vitro, gEGF significantly promoted UMNSAH/DF-1 (DF-1) cell proliferation, whereas both gTFF2 and gEGF enhanced epithelial cell migration. In vivo, oral administration of recombinant *B. subtilis* significantly reduced mortality from 75.0% and 66.7% in the PBS and pHT43 groups to 50%, 33.3%, and 25.0% in the gTFF2, gEGF, and gTFF2+gEGF groups, respectively. Cecal colonization of *S. pullorum* was reduced by 2–4 log_10_ CFU/g, accompanied by improved average daily gain and immune organ indices. Treatment also significantly decreased serum IL-6 levels and increased TGF-β levels (*p* < 0.05), indicating attenuation of the inflammatory response. Histological analysis showed significantly increased villus height (0.94–1.58-fold) and villus height-to-crypt depth ratio (1.47–3.25-fold) compared with the infected controls, together with markedly alleviated hepatic and intestinal lesions. The combined administration of gTFF2- and gEGF-expressing strains consistently exhibited the greatest protective efficacy. Collectively, these findings demonstrate that engineered probiotic-mediated delivery of mucosal repair factors represents a promising antibiotic-alternative strategy for preventing pullorum disease and improving intestinal health in poultry production.

## 1. Introduction

The digestive system of newly hatched chicks is relatively fragile and highly vulnerable to nutritional, environmental, and physiological stressors. Intestinal health is essential not only for nutrient digestion and absorption, but also for maintaining mucosal barrier integrity and immune defense against enteric pathogens [1]. Among enteric bacterial diseases, pullorum disease caused by *Salmonella pullorum* (*S. pullorum*) remains an important threat to poultry production, particularly in young chicks with immature intestinal and immune systems. *S. pullorum* can be transmitted both vertically, from infected breeder hens to their offspring through contaminated eggs, and horizontally through direct contact with infected birds, feces, contaminated feed, water, or the environment [2]. Moreover, surviving or asymptomatic carrier birds may develop persistent infections and continuously shed the pathogen, serving as an important reservoir for transmission within poultry flocks and making disease eradication particularly challenging [3]. Infected chicks typically exhibit diarrhea, anorexia, depression, impaired growth, and high mortality. In addition to acute mortality, *S. pullorum* infection can severely disrupt intestinal barrier integrity and early intestinal development, thereby impairing nutrient absorption and reducing subsequent production performance. Traditionally, antibiotics have been widely used in feed to improve gut microbiota and control bacterial infections in chicks [4,5]. However, the extensive use of antibiotics has accelerated the emergence of antimicrobial resistance and disrupted microbial ecological balance [6]. Furthermore, increasing restrictions on antibiotic use in animal production have intensified the demand for effective antibiotic alternatives that can enhance intestinal resilience and reduce pathogen colonization in young chicks [7]. Among the available alternatives, Bacillus subtilis (B. subtilis) has attracted considerable attention as a food-grade probiotic with excellent environmental adaptability and gastrointestinal survival capacity [8] or recombinant delivery of bioactive molecules, with broad application potential in livestock and poultry production. [9,10,11].

Some bioactive substances have been shown to improve intestinal morphology and function [12,13,14]. Trefoil factors (TFFs) are a class of mucin-related peptides containing one or more trefoil domains [15]. TFF2 was first isolated as a side component of porcine insulin production and is recognized for roles in mucosal repair, defense, and expansion, particularly in the digestive and respiratory systems [16]. In veterinary science, TFF2 has been shown to promote healing of gastric ulcers in mice [17]. In piglets, weaning induces increased TFF2 expression, highlighting its critical role in maintaining mucosal integrity [18,19].

Epidermal growth factor (EGF) is a 53-amino-acid peptide primarily synthesized in the salivary glands and kidneys. EGF is heat- and acid-stable, resistant to protease digestion [20], and has a wide range of biological activities in the intestinal epithelium, including stimulation of intestinal epithelial cell proliferation, differentiation, and intestinal maturation [21]. EGF can also reduce the colonization by intestinal pathogens. For example, it decreased *Campylobacter jejuni* colonization and prevented claudin-4 disruption in chicks [22], and it protected tight junctions in mice infected with *Clostridioides difficile* [23].

Given the important roles of TFF2 and EGF in intestinal mucosal repair and epithelial regeneration, we hypothesized that an engineered *B. subtilis* strain expressing gTFF2 or gEGF could enhance intestinal health and reduce the severity of *S. pullorum* infection in chicks. In this study, we constructed two recombinant *B. subtilis* strains and to evaluate their prophylactic effects on growth performance, intestinal injury, bacterial colonization, and inflammatory responses in a chick model of pullorum disease.

## 2. Materials and Methods

### 2.1. Bacterial Strains, Plasmid, Primers, and Media for Bacterial Growth

The bacterial strains, vectors, and oligonucleotides used in this study are listed in Table 1*. B. subtilis* WB800N and shuttle vector pHT43-His were used as the expression host and expression vector, respectively (both purchased from Miaoling Biotechnology, Wuhan, China). *Escherichia coli* (*E. coli*) DH5α (Sangon Biotech, Shanghai, China) was used for subcloning and plasmid amplification. *S. pullorum* CVCC528 (preserved in our laboratory) was used to establish an animal infection model. The identity of the bacterial isolate was confirmed by 16S rRNA gene sequencing together with PCR amplification of the Salmonella-specific invA gene prior to the animal experiments [24]. All of these bacteria were cultured in Luria–Bertani (LB) culture medium (1% *w*/*v* of peptone, 0.5% *w*/*v* of yeast extract and 0.5% *w*/*v* of sodium chloride) at 37 °C with shaking. Kanamycin and chloramphenicol were both added at 10 μg/mL when necessary.

### 2.2. Plasmid Construction and Transformation

The intestinal tissues were collected from 12 day-old chick embryos under sterile conditions. Tissues were washed three times with sterile PBS, flash-frozen in liquid nitrogen, and ground in a pre-chilled mortar. Total RNA was extracted from the intestinal tissue using TRIzol reagent (TIANGEN BIOTECH, Beijing, China) following the protocol described in the cited reference [25], and cDNA was generated using a reverse-transcription kit (TaKaRa, Dalian, China). Gene fragments for gTFF2 and gEGF were amplified by PCR using primers gTFF2-F/R and gEGF-F/R, designed from NCBI sequences (gTFF2: KC590328.1; gEGF: NM_001001292.1). PCR products were cloned into the *Bam*H I/*Sma* I of vector pHT43-His, generating pHT43-gTFF2 and pHT43-gEGF. The engineered plasmids were transformed into *E. coli* DH5α and selected on ampicillin (50 µg/mL). Ligation was confirmed by restriction digestion and PCR. Then, the recombinant plasmids and the empty plasmid were transformed into *B. subtilis* strain WB800N [26], and selected on LB plates with chloramphenicol (10 µg/mL). Positive colonies were confirmed using colony PCR, yielding pHT43-gTFF2/WB800N, pHT43-gEGF/WB800N, and pHT43/WB800N.

### 2.3. Expression of Recombinant gEGF and gTFF2 Protein in B. subtilis

For assessing gene expression in *B. subtilis* WB800N, three recombinant strains were incubated overnight in 5 mL of LB medium supplemented with 10 μg/mL of chloramphenicol at 37 °C, 200 rpm. Subsequently, a 0.2 mL sample from the overnight culture (1% *v*/*v*) was transferred into 20 mL of fresh LB with chloramphenicol and grown under the same conditions. When the culture reached OD_600_ of 0.7–0.8, 1 mM of isopropyl β-D-1-thiogalactopyranoside (IPTG) was added to induce expression (t = 0). The pHT43 vector encodes an α-amylase signal peptide that directs secretion of the fused protein upon induction. After a 12 h induction period, cultures were centrifuged at 12,000 rpm for 10 min at 4 °C, and supernatants were collected for analysis, with the pHT43/WB800N serving as a control.

The expression conditions were optimized using one-factor-at-a-time technique to maximize the gTFF2 and gEGF production at different levels of IPTG concentration (0.2, 0.5, 1 and 2 mM), temperature (25, 30, 37 and 42 °C), and induction duration (6, 12, 18, 24, 30, 36 and 42 h). Cultures were induced at OD_600_ = 0.7–0.8 and centrifuged as described above. The supernatant and bacterial pellets were separately collected and stored at −40 °C for subsequent Western blot analysis.

### 2.4. In Vitro Experiment

Protein samples were collected from 50 mL of the recombinant *B. subtilis* cultures following 18 h of induction at 37 °C with shaking. Cultures (20 mL) were centrifuged at 12,000 rpm for 2 min at room temperature (RT) and the pellets were resuspended in 2 mL PBS. After sonication, the lysates were centrifuged at 12,000 rpm for 2 min at RT. The supernatant was collected and filtered through 0.22 μm filters. The total protein concentration in the collected supernatant was measured using a NanoDrop spectrophotometer (Thermo Fisher Scientific, Waltham, MA, USA).

UMNSAH/DF-1 (DF-1) chicken embryo fibroblast cells (preserved in our laboratory) were used for in vitro experiments. DF-1 is an established chicken embryo fibroblast cell line widely used in avian research and available through ATCC (CRL-3586). Cells were cultured in Dulbecco’s modified Eagle’s medium (DMEM; Thermo Fisher Scientific, Waltham, MA, USA) supplemented with 10% fetal bovine serum at 37 °C in a humidified atmosphere containing 5% CO_2_.

#### 2.4.1. Cell Proliferation

Confluent cells were trypsinized and seeded into 96-well plates (100 μL/well), followed by incubation at 37 °C with 5% CO_2_ for 12 h. The cell cultures were washed with 100 µL PBS three times and starved for 12 h in DMEM without fetal bovine serum. Protein samples were prepared as twofold serial dilutions in sterile PBS and added to the wells in 100 μL volumes, yielding final concentrations of 0.5, 1, 2, and 4 ng/μL. The cultures were maintained for 12 h. Subsequently, 10 µL Cell Counting Kit-8 (Sangon Biotech Co., Ltd., Shanghai, China) was added to the cell cultures and incubated for 1.5 h. The OD_450_ of cultures was measured using a microplate reader (Thermo Electron Corporation, Waltham, MA, USA).

#### 2.4.2. Cell Migration

Confluent cells were trypsinized and seeded into 6-well plates (2 mL/well) and incubated at 37 °C with 5% CO_2_ for 24 h to reach a confluent monolayer. The culture medium was discarded, and the cell monolayer was scratched with a cross-shaped wound in each well, according to a previous study [27]. Each well was then washed three times with 200 µL of sterile PBS. The protein samples were diluted in the culture medium, and 200 μL was added to each well to achieve final concentrations of gTFF2 at 2 ng/μL and gEGF at 0.5 ng/μL. Three parallel samples were prepared for each condition. Cell migration was observed using an inverted microscope (Leica DM3000, Leica Microsystems, Wetzlar, Germany) at 0, 36, and 72 h. The extent of wound closure was quantified by measuring the area of the scratch that had been covered by migrating cells, using the ImageJ software (version 1.54f). Wound closure = $\left[\frac{A_{t=0h}-A_{t=\Delta h}}{A_{t=0h}}\right]\times 100\%$.

### 2.5. In Vivo Experiment

#### 2.5.1. Animals, Housing and Diets

A total of 72 5 day-old specific-pathogen-free (SPF) chicks were obtained from the Experimental Animal Center of the Harbin Veterinary Institute, Chinese Academy of Agricultural Sciences. During the experiment, all chicks were housed in individual cages and had access to water and feed. A corn-soybean-meal basal diet in mash form was formulated to meet the nutrient requirements of chicks, as per the *Feeding Standard of Chicken* (NY/T 33-2004) [28] (Table 2).

#### 2.5.2. Immunization and Challenge in Chicks

**Figure 1:**
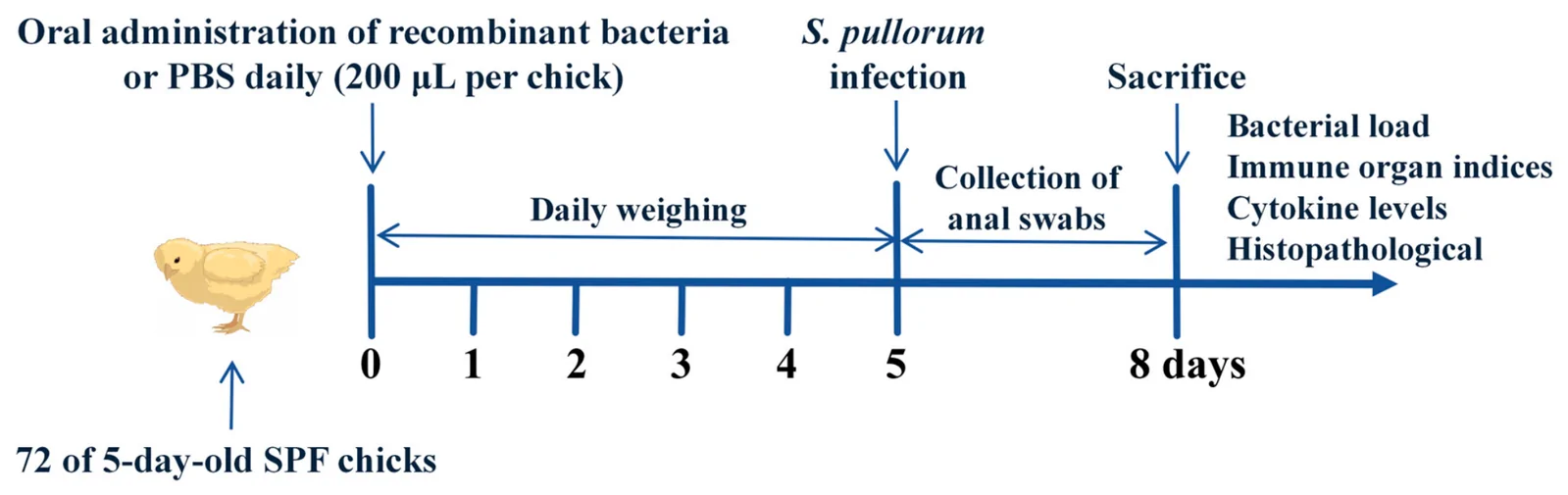
Procedure of oral immunization, infection and sampling. Following five consecutive days of oral immunization, the chicks were challenged with *S. pullorum*.

Chicks were randomly divided into six groups (12 per group) and subjected to different treatments for 8 d, implicitly assuming a gender-neutral distribution. Healthy chicks were maintained under standard conditions and served as the blank control group. The remaining groups of chicks were orally gavaged once daily with 200 μL of the following treatments: sterile PBS (the PBS group), pHT43/WB800N culture (the pHT43 group), pHT43-gTFF2/WB800N culture (the gTFF2 group), pHT43-gEGF/WB800N culture (the gEGF group), or a combination of pHT43-gTFF2/WB800N and pHT43-gEGF/WB800N cultures (the gTFF2+gEGF group, 100 μL each). Recombinant *B. subtilis* cultures induced for 18 h were collected and adjusted to 5 × 10^9^ CFU/mL using sterile PBS. Five days later, all chicks except those in the blank control group were orally administered with *S. pullorum* CVCC528 (1 × 10^8^ CFU/chick), as determined in previous studies [29,30]. A 3 d observation period was conducted, during which three chicks from each group were randomly selected daily to collect anal swabs. After 3 days of treatment, three chicks randomly selected from each group were deeply anesthetized with isoflurane (3–5%) and subjected to terminal cardiac blood collection. Blood was collected by cardiac puncture, and euthanasia was completed by exsanguination under anesthesia. Following euthanasia, all chicks were subjected to a systematic necropsy. Gross pathological lesions of the liver, spleen, intestine, cecum, and other major organs, including congestion, hemorrhage, necrosis, organ enlargement, and intestinal lesions, were examined and recorded. Serum, cecal contents, and a portion of the ileal tissue were then collected for subsequent analyses, as illustrated in Figure 1.

Because the intervention was based on live recombinant *B. subtilis* rather than purified recombinant proteins, the administered dose was standardized according to bacterial cell number. The absolute amount of secreted gTFF2 or gEGF per dose was not determined, because the culture supernatant contained multiple secreted bacterial proteins and the recombinant factors were not purified prior to oral administration.

#### 2.5.3. Growth Performance and Clinical Symptoms

During the experiment, all chicks were weighed on an empty stomach at 07:00 each day, and body weight (BW) was documented. ADG (Average Daily Gain) = end weight measurement − beginning weight measurement/measurement day. Clinical signs and mortality were observed every day. Mortality rate (%) = number of dead chicks per group/number of chicks per group × 100%.

#### 2.5.4. Real-Time Fluorescence Quantitative PCR (qPCR) Analysis

The anal swabs collected were soaked in 1 mL PBS at 4 °C overnight, and the supernatant was acquired by centrifugation to detect the *S. pullorum* quantification. The bacterial loads of the anal swabs were triplicated and determined via qPCR using an ABI Prism 7500 sequence detection system (Applied Biosystems, Foster City, CA, USA). Before the assay, a standard curve was established for absolute quantification of the bacteria. The standard plasmid carrying the InvA gene (NCBI, Accession Number: M90846.1) at an initial concentration of 1 × 10^8^ copies/μL was diluted 10-fold, and each dilution was repeated five times for qPCR. Using the logarithm of the dilution of the plasmid standard as the x-axis and the corresponding cycle threshold (Ct) value as the y-axis, a quantitative standard curve corresponding to the plasmid copy number and Ct value was constructed. The Ct value of the measured sample was used to calculate the copy number of *S. pullorum* using a standard curve.

#### 2.5.5. Bacterial Cell Counting

Three days after infection, three randomly chosen chicks from each group were subjected to cardiac blood sampling. The cecal contents were also collected and weighed using a sterile 2 mL Eppendorf tube, ranging from 0.05 to 0.15 g. The collected samples were diluted with sterile PBS at a 1:100 volume ratio and homogenized twice at 3200 rpm for 10 min using a homogenizer (Bertin Technologies, Montigny-le-Bretonneux, France). After gradient dilution to 10^−2^–10^−8^, 10 μL of the dilutions were plated on BS agar medium (Qingdao Hope Bio-Technology Co., Ltd, Qingdao, China). The plates were inverted and incubated at 37 °C for 16–24 h to allow for colony growth. The original bacterial load of each sample was calculated based on the obtained colony counts.

#### 2.5.6. Detection of Immune Organ Indices

The spleen, thymus, and bursa of Fabricius were collected from three chicks. The organs were thoroughly rinsed to remove any blood residue and weighed to obtain accurate measurements. The immune organ index was calculated as follows: immune organ index (g/kg) = immune organ weight (g)/live weight of chicks (kg).

#### 2.5.7. Cytokine Levels in Blood Serum

On the third day post-infection, three chicks per group were sacrificed to collect blood from their hearts for serum preparation. Cytokine levels in serum were detected using ELISA kits (MeiMian Industrial Co., Ltd., Yancheng, China) following the manufacturer’s instructions. Types of detection included interleukin-6 (IL-6), tumor necrosis factor (TNF-α), and transforming growth factor-β (TGF-β), and the cytokine concentrations were analyzed by drawing a standard curve to calculate the numerical values for each ELISA plate. Each sample was replicated three times, and blank wells were used as controls.

#### 2.5.8. Histopathological Analysis

Tissue blocks from the liver and ileum were preserved in 10% neutral buffered formalin for 24 h, processed into paraffin wax, and subsequently sliced into sections 5 µm thick. These sections were subjected to staining with hematoxylin and eosin (HE), enabling the observation and analysis of morphological alterations through a light microscopy. Each sample was examined using three non-consecutive sections to ensure the selection of representative fields with intact and aligned villi. Representative H&E-stained sections from each experimental group were assessed using a semi-quantitative histopathological scoring system (0–4) based on lesion severity. The detailed scoring criteria for liver and ileum are shown in Table 3. Subsequently, the cutting-edge software Motic Image 2000 (https://www.motic.com; accessed on 20 July 2026) was used to quantify and analyze essential parameters, including the length of the small intestinal villi, the depth of crypts, and the villus length-to-crypt depth (V/C) ratio.

#### 2.5.9. Statistical Analysis

Analyses were performed in SPSS (27.0.1.0) and presented as mean ± SD. All data were analyzed using one-way ANOVA to detect overall differences among treatment groups, and post hoc pairwise comparisons were conducted using Duncan’s multiple range test. Two pre-specified contrasts were evaluated: (i) versus PBS; (ii) versus vector control (pHT43). Statistical significance was set at *p* < 0.05.

## 3. Results

### 3.1. Construction and Characterization of Recombinant B. subtilis

**Figure 2:**
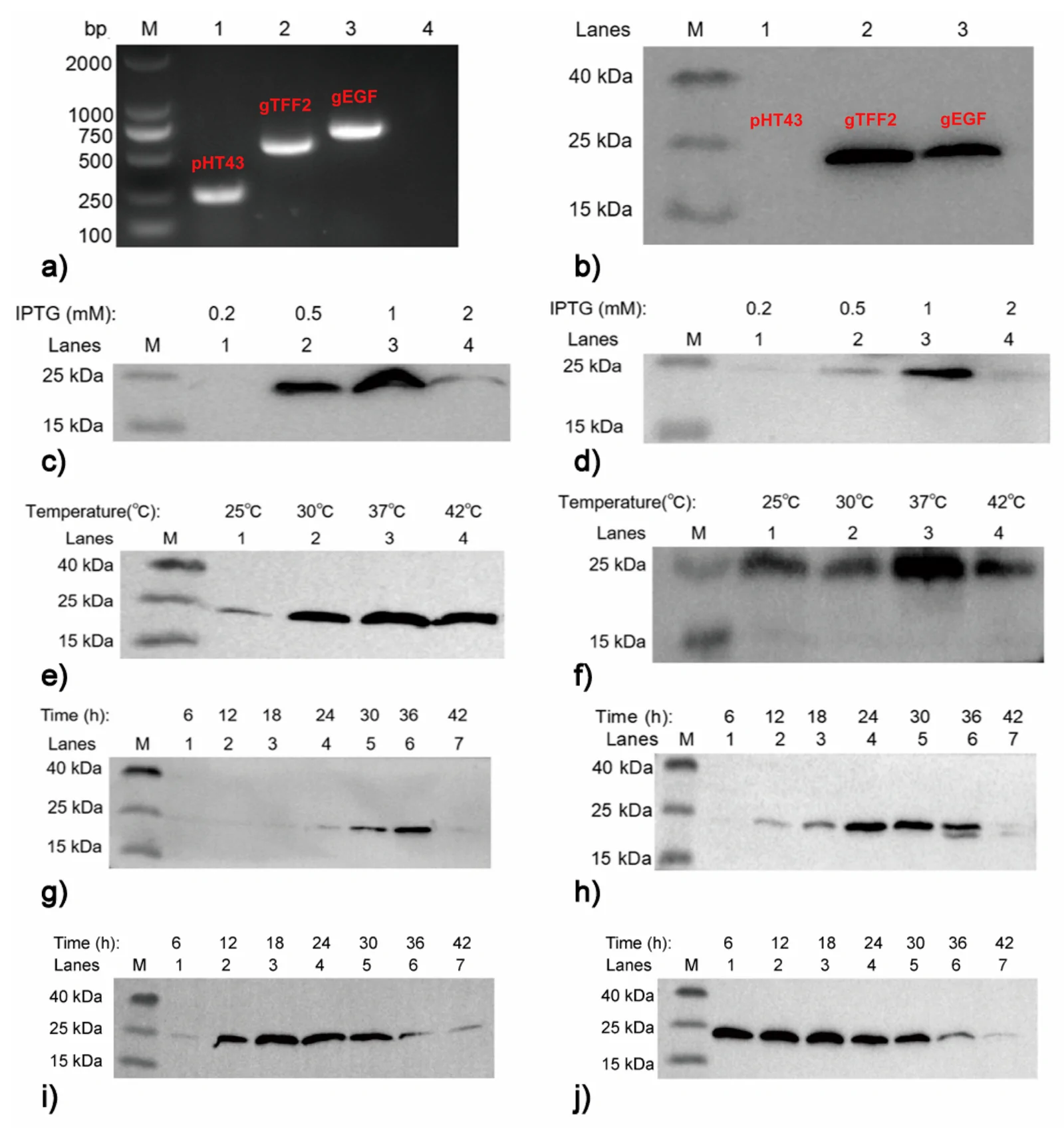
Construction and expression of recombinant plasmid. (**a**) PCR identification of transformants. Lane 1, pHT43/WB800N. Lane 2, pHT43-gTFF2/WB800N. Lane 3, pHT43-gEGF/WB800N. Lane 4, negative control. (**b**) Molecular weight analysis of gTFF2 and gEGF protein. Lane 1, the supernatant samples of pHT43/WB800N. Lane 2, the supernatant samples of pHT43-gEGF/WB800N. Lane 3, the supernatant samples of pHT43-gTFF2/WB800N. (**c**,**d**) The supernatant samples of pHT43-gTFF2/WB800N and pHT43-gEGF/WB800N at the IPTG concentration of 0.2, 0.5, 1 and 2 mM. (**e**,**f**) The supernatant samples of pHT43-gTFF2/WB800N and pHT43-gEGF/WB800N were taken 24 h post-induction at 25, 30, 37 and 42 °C. (**g**,**h**) The supernatant samples of pHT43-gTFF2/WB800N and pHT43-gEGF/WB800N were taken every 6 h post-induction. (**i**,**j**) The bacterial pellets of pHT43-gTFF2/WB800N and pHT43-gEGF/WB800N were taken every 6 h post-induction.

We successfully amplified gTFF2 and gEGF gene fragments from the chicken genome using PCR, generating specific bands of approximately 400 bp (gTFF2) and 500 bp (gEGF) in length, respectively (Figure S1). The amplified gTFF2 and gEGF fragments were cloned into pHT43. The recombinant plasmids were confirmed via PCR and restriction digestion using *Bam*H I and *Sma* I (Figure S2). After sequence verification, the recombinant plasmids (pHT43-gTFF2, pHT43-gEGF) and empty plasmid (pHT43) were transformed into WB800N strains. Colony PCR confirmed the insertion of the three plasmids into *B. subtilis* WB800N (Figure 2a).

Next, the expression of gTFF2 and gEGF was investigated using the pHT43 vector as a control. When the culture reached an OD600 of 0.6–0.8, 1 mM IPTG was added to induce protein expression, and the supernatant was collected 12 h post-induction. Western blot analysis confirmed extracellular protein expression (Figure 2b). Protein expression optimization revealed that the optimal IPTG concentration was 1 mM (Figure 2c,d), and the optimal induction temperature for both target proteins was 37 °C (Figure 2e,f). The secretion of gTFF2 reached its maximum after 36 h of induction (Figure 2g), whereas the secretion of gEGF reached its maximum after 24 h of induction (Figure 2h). Intracellular gTFF2 and gEGF both reached relatively high expression levels at 18 h post-induction (Figure 2i,j).

### 3.2. gEGF Promotes Proliferation of DF-1 Cells

**Figure 3:**
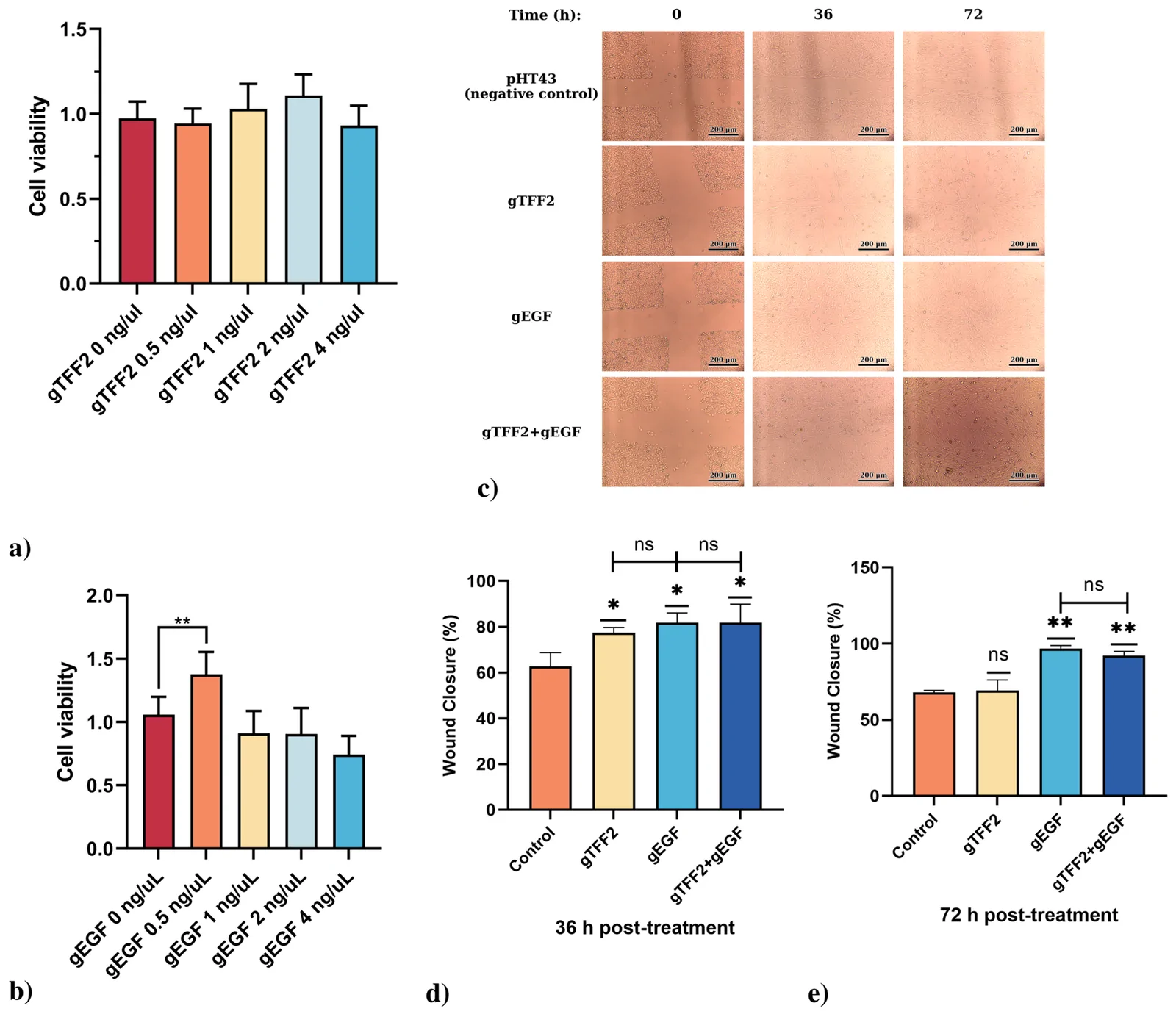
The analysis of the proliferation and migration effect of gTFF2 and gEGF on DF-1 cells. Wound closure is expressed as the remaining area uncovered by the cells. (**a**,**b**) Effects of different concentrations of protein on the proliferation of DF-1 cells. All experiments were performed in triplicate, and each bar represents the mean value. N = 6, ** *p* < 0.01. (**c**) The analysis of the migration effect of gTFF2 and gEGF on DF-1 cells. By 72 h of treatment, the scratch wounds in the gEGF group and gTFF2+gEGF group were almost completely healed. (**d**) Analysis of the wound closure rate for all groups in 36 h post-treatment. (**e**) Analysis of the wound closure rate for all groups in 72 h post-treatment. All experiments were independently performed in triplicate, and each bar represents the mean value. N = 3, * p < 0.05, ** p < 0.01, * means difference analysis with negative control.

To evaluate the effects of gTFF2 and gEGF on cell proliferation, the cell culture supernatants were quantified, serially diluted, and incubated with starved DF-1 cells. After 12 h, cell viability was assessed using the CCK-8 method. The results demonstrated that gTFF2 at various concentrations did not significantly promote DF-1 cell proliferation (Figure 3a), whereas gEGF significantly enhanced DF-1 cell proliferation at a concentration of 0.5 ng/μL (*p* < 0.01) (Figure 3b).

### 3.3. Both gTFF2 and gEGF Promote Migration of DF-1 Cells

The effects of gTFF2 and gEGF on the migration of DF-1 cells were analyzed using a cell scratch assay. The diluted protein samples were added to DF-1 cells that had been subjected to a cross-shaped scratch, and cell migration was monitored over a 72 h period. As shown in Figure 3c, after 36 h of treatment, the scratch wounds in the gTFF2, gEGF, and gTFF2+gEGF groups began to heal, with more pronounced healing observed in the gEGF and gTFF2+gEGF groups. After 72 h of treatment, the scratch wounds in the gEGF and gTFF2+gEGF groups healed almost completely. As shown in Figure 3d,e, compared to the negative control, all three groups showed significant enhancement of cell migration within 36 h (*p* < 0.05), and both the gEGF group and the gTFF2+gEGF exhibited nearly 100% wound healing within 72 h (*p* < 0.01). However, the synergistic effect of their combined application did not result in superior enhancement of cell migration.

### 3.4. Oral gTFF2/gEGF-Expressing Recombinant B. subtilis Enhances Growth Performance in Chicks

Chicks were randomly divided into six groups and were orally administered different solutions daily for 5 days prior to *S. pullorum* infection. The changes in the chick BW and ADG are shown in Table 4. On day 1, no significant difference in BW was found among these groups. On day 6, BW of the chicks in the pHT43 and gTFF2 groups increased slightly, but there were no significant differences compared with the PBS and blank control group (*p* > 0.05). However, BW and ADG in the gEGF and gTFF2+gEGF groups were significantly higher (*p* < 0.05) and highest in the gTFF2+gEGF group. Interestingly, while BW and ADG in the gTFF2, gEGF, and gTFF2+gEGF groups increased compared to the pHT43 group, the differences were not statistically significant. This suggests that *B. subtilis* itself may also play a role in promoting the growth and development of chicks.

### 3.5. Oral gTFF2/gEGF-Expressing Recombinant B. subtilis Reduces Mortality in Chicks Challenged with S. pullorum

**Figure 4:**
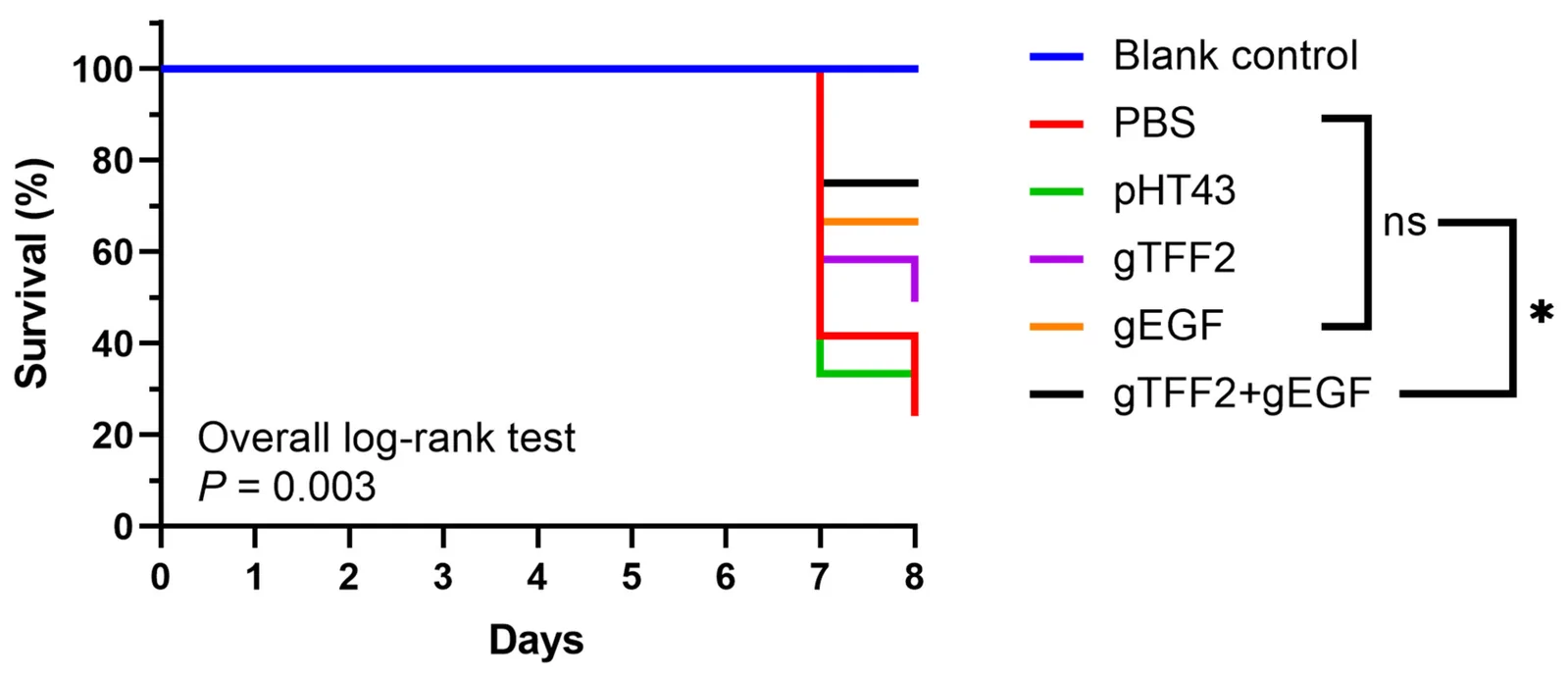
The survival (%) in different treatment groups of chicks. * indicates a statistically significant difference (*p* < 0.05).

To exclude potential confounding factors (e.g., stress and congenital diseases), we systematically monitored the baseline status of chicks in all experimental groups from day 1 to day 6 prior to pathogen challenge, ensuring consistency in water intake, feeding patterns, behavioral activity, and absence of adverse reactions. From day 6 to 8 after *S. pullorum* challenge, the chicks exhibited symptoms of depression, anorexia, white feces, and often huddled together. Additionally, the mortality of the chicks was recorded daily, and the mortality rate was calculated at the end of the experimental period (Figure 4). The mortality rate was 75% in the PBS group and 66.67% in the pHT43 group, and compared with both groups, the mortality rates in the gTFF2, gEGF, and gTFF2+gEGF groups were significantly reduced, with rates of 50%, 33.33%, and 25.00%, respectively. These results indicate that oral administration of recombinant *B. subtilis* expressing gEGF and gTFF2 effectively protects against mortality caused by *S. pullorum*. During the experiment, chicks in the blank control group showed normal performance without adverse reactions.

### 3.6. Oral gTFF2/gEGF-Expressing Recombinant B. subtilis Reduces Fecal Bacterial Load in Chicks

**Figure 5:**
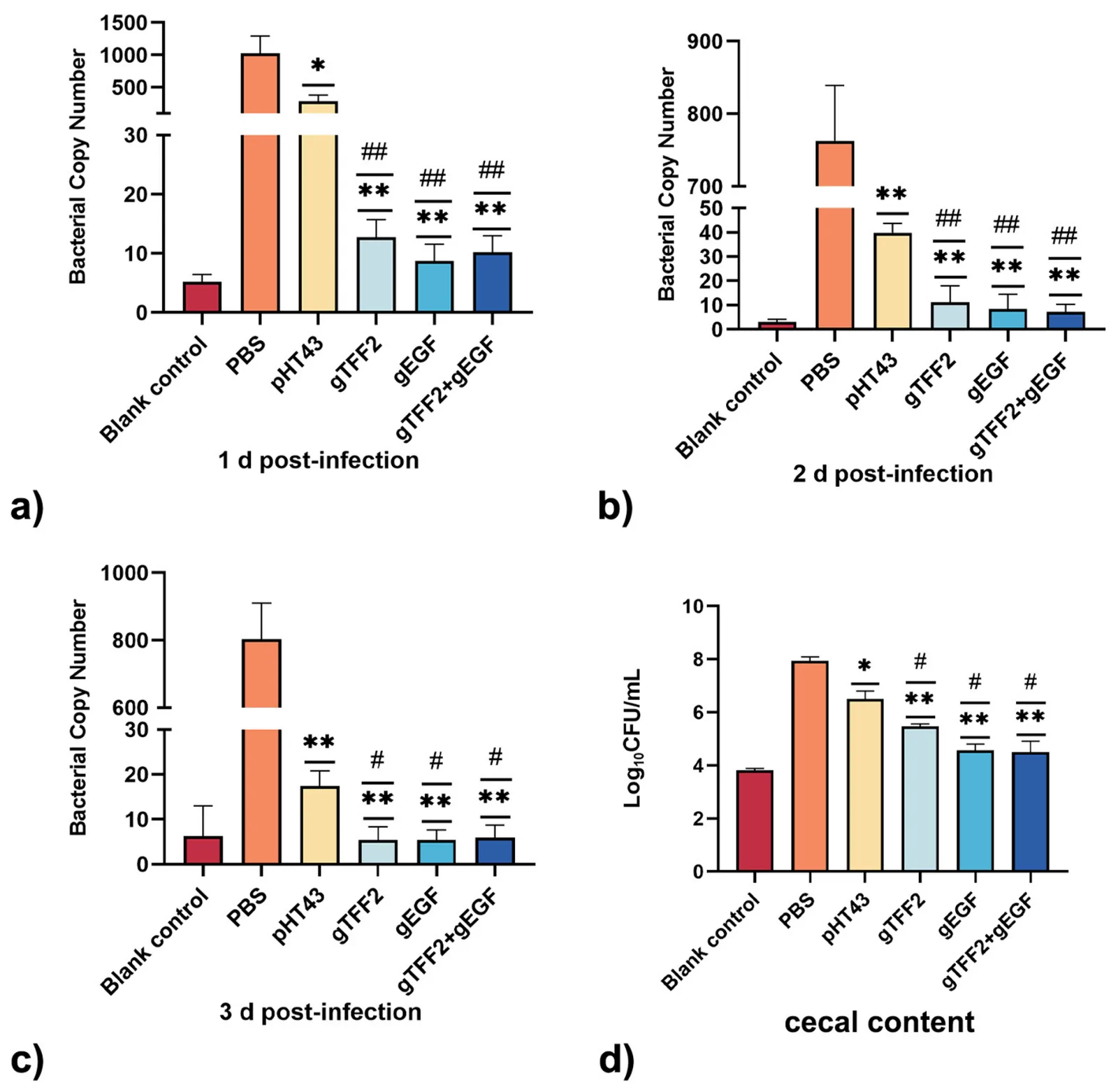
The impact of recombinant *B. subtilis* on infection by *S. pullorum*. (**a**–**c**) Detection of copy numbers of *S. pullorum* in anal swabs of the different groups of chicks at 1–3 days post-infection (dpi). (**d**) Average logarithm counts of *S. pullorum* in the cecal contents of the different groups of chicks. All experiments were independently performed in triplicate, and each bar represents the mean value. N = 3, * p < 0.05, ** p < 0.01, * means difference analysis with PBS group as control; *# p* < 0.05, *## p* < 0.01, # means difference analysis with pHT43 group as control.

The bacterial load in the anal swabs was detected via qPCR at 1, 2, and 3 d post-infection (Figure 5a–c). Compared to the PBS group, the bacterial load in anal swabs was reduced by approximately two orders of magnitude in the gTFF2, gEGF, and gTFF2+gEGF groups, with extremely significant differences (** *p* < 0.01). Compared with the pHT43 group, the gTFF2, gEGF, and gTFF2+gEGF groups also exhibited lower bacterial loads, with a 10- to 50-fold reduction on the 1st and 2nd day (## *p* < 0.01) and about a 2-fold reduction on the 3rd day *(# p* < 0.05). These findings suggest that recombinant strains markedly decreased the early infection burden of *S. pullorum*.

### 3.7. Oral gTFF2/gEGF-Expressing Recombinant B. subtilis Inhibits the Colonization of S. pullorum in the Intestine

The enumeration of *S. pullorum* in the cecal contents was performed using the plate counting method. The results showed that, compared with the PBS group, the bacterial count in cecal contents was reduced by 2 to 4 orders of magnitude in the gTFF2, gEGF and gTFF2+gEGF groups (** *p* < 0.01). In addition, compared with the pHT43 group, a 1- to 2-log reduction was observed in the same groups (# *p* < 0.05) (Figure 5d). These findings indicated that oral administration of recombinant *B. subtilis* effectively inhibited the colonization of *S. pullorum* in the intestine.

### 3.8. Oral gTFF2/gEGF-Expressing Recombinant B. subtilis Improves Immune Organ Indices in Chicks

The spleen, thymus, and bursa of Fabricius are important immune organs in chicks, and organ indices reflect the maturity of the immune system. The changes in immune organ indices among the different groups were shown in Table 5. Compared with the PBS and pHT43 groups, chicks in the gTFF2 and gEGF groups had significantly higher spleen and thymus indices (*p* < 0.05). The gTFF2+gEGF group showed an extremely significant increase in the spleen (*p* < 0.01) and a significant increase in thymus indices *(p* < 0.05). There were no significant differences in the bursa of Fabricius index between groups. These results indicated that both gTFF2 and gEGF promoted the development of the thymus and spleen in chicks and that their combined use could effectively promote the development of the chick spleen.

### 3.9. Oral gTFF2/gEGF-Expressing Recombinant B. subtilis Alleviates Inflammatory Responses in Chicks

**Figure 6:**
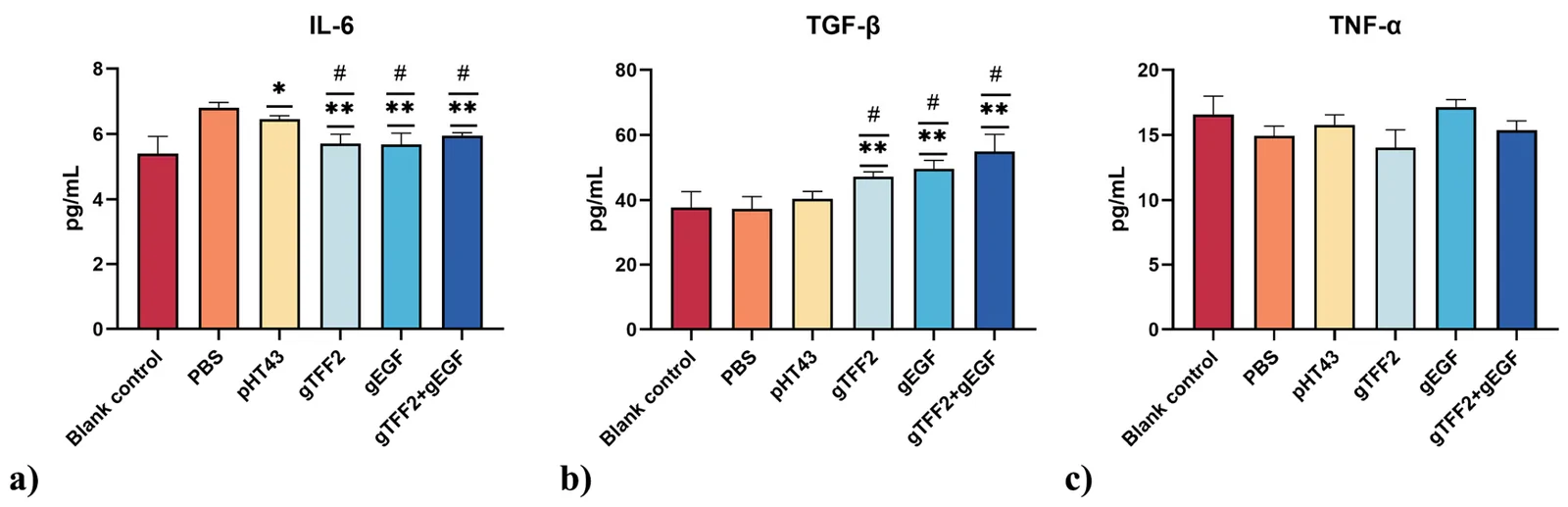
Comparison of relative serum cytokine levels among different treatment groups of chicks. ELISA was used to determine cytokine levels. (**a**) IL-6; (**b**) TGF-β; (**c**) TNF-α. All experiments were independently performed in triplicate, and each bar represents the mean ± SD (N = 3). * *p* < 0.05, ** *p* < 0.01 versus the PBS group; # *p* < 0.05 versus the pHT43 group.

*S. pullorum* can induce an inflammatory response in the body. In response, the immune system produces cytokines to counteract the pathogen-induced inflammation. Based on the standard curve plotted using the standard samples in the ELISA kit, we measured the protein expression levels of the pro-inflammatory cytokines IL-6 and TNF-α, as well as the anti-inflammatory cytokine TGF-β, as shown in Figure 6. Relative to the PBS group, the gTFF2, gEGF, and combination groups exhibited significantly lower IL-6 and higher TGF-β levels (** *p* < 0.01). A similar trend was observed compared to the pHT43 group, with significant modulation of both cytokines (# *p* < 0.05). There were no significant differences in the levels of TNF-α among the groups.

### 3.10. Oral gTFF2/gEGF-Expressing Recombinant B. subtilis Provides Protective Effects on Liver and Intestine in Chicks

**Figure 7:**
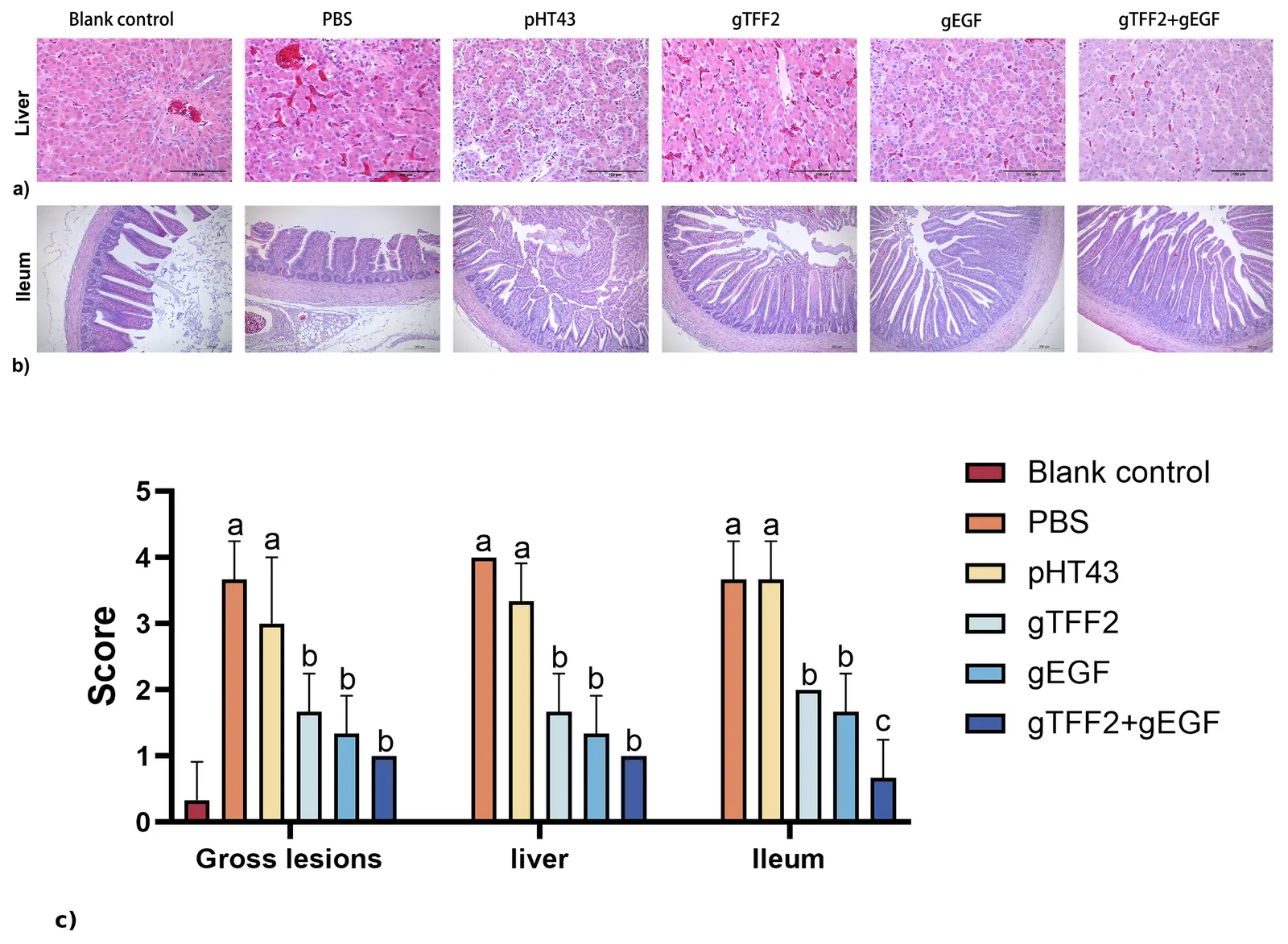
Histopathological examination of the liver and ileum in chicks following *S. pullorum* infection. (**a**) Representative hematoxylin and eosin (H&E)-stained sections of the liver. Chicks in the PBS and pHT43 groups exhibited marked hepatocellular degeneration and focal necrosis, characterized by detachment from surrounding cells and the presence of cytoplasmic vacuoles of varying sizes. In contrast, the gTFF2, gEGF, and gTFF2+gEGF groups showed markedly attenuated hepatic lesions, characterized by reduced inflammatory infiltration and a more preserved hepatic architecture. The gTFF2+gEGF group exhibited liver morphology most closely resembling that of the blank control group. (**b**) Representative H&E-stained sections of the ileum. Severe intestinal lesions were observed in the PBS and pHT43 groups, including villus disruption, epithelial cell shedding, crypt damage, and inflammatory cell infiltration within the lamina propria. These pathological changes were markedly alleviated in the gTFF2-, gEGF-, and gTFF2+gEGF-treated groups, which displayed better preservation of villus structure, more intact intestinal mucosa, and reduced inflammatory infiltration. Scale bars: 100 μm (liver) and 200 μm (ileum). (**c**) Gross lesion scores and histopathological scores of the liver and ileum in different treatment groups. Treatment with gTFF2 and/or gEGF reduced lesion scores compared with the PBS group, with the combined treatment showing the lowest scores. Different lowercase letters indicate statistically significant differences among groups (*p* < 0.05).

To assess the protective effects of recombinant *B. subtilis* against *S. pullorum* infection, histopathological changes in the liver and ileum of chicks were evaluated. The livers of chicks in the PBS and pHT43 groups exhibited extensive hepatocellular degeneration and focal necrosis, characterized by detachment from surrounding cells and the presence of cytoplasmic vacuoles of varying sizes. Additionally, a substantial infiltration of inflammatory cells was observed in the liver tissue. In contrast, the livers of chicks in the gTFF2, gEGF, and gTFF2+gEGF groups exhibited milder pathological changes and reduced inflammatory infiltration, with more orderly hepatocyte arrangement. Notably, liver histological sections from the gTFF2+gEGF group exhibited a morphology more closely resembling that of the blank control group, suggesting that the combined administration of gTFF2 and gEGF may have a superior therapeutic effect (Figure 7a). In the ileum of chicks in the PBS and pHT43 groups, there were observed disruptions in the villi, extensive shedding of epithelial cells, significant damage to crypts, and notable infiltration of inflammatory cells into the lamina propria. However, in the gTFF2, gEGF, and gTFF2+gEGF groups, the lesions were milder with fewer villous ruptures and relatively intact mucosal structures (Figure 7b). Semi-quantitative histopathological scoring further confirmed the protective effects of recombinant *B. subtilis* (Figure 7c). The PBS and pHT43 groups exhibited the highest histopathological scores in both the liver and ileum, indicating severe tissue injury. In contrast, the gTFF2, gEGF, and gTFF2+gEGF groups showed significantly lower lesion scores (*p* < 0.05). Among these, the combined gTFF2+gEGF treatment group exhibited the lowest histopathological scores, approaching those of the blank control group.

The lengths of the small intestine villi, crypt depth, and V/C ratio were analyzed in Table 6. The gTFF2 and gEGF groups showed a significant increase in villus length, ranging from 0.94- to 1.19-fold (*p* < 0.05), with the greatest increase observed in the gTFF2+gEGF group, which reached 1.58-fold (*p* < 0.01). In terms of crypt depth, no significant differences were observed among the gTFF2, gEGF, and gTFF2+gEGF groups, whereas all treatment groups showed markedly reduced crypt depth compared to both control groups (*p* < 0.05). Furthermore, V/C ratio was improved by 1.47- to 2.56-fold in the gTFF2 and gEGF groups (*p* < 0.05), and further elevated to 2.29- to 3.25-fold in the gTFF2+gEGF group (*p* < 0.01). These results indicated that recombinant *B. subtilis* alleviates intestinal villus damage. Moreover, the combined use of these treatments resulted in pronounced effects.

## 4. Discussion

*S. pullorum* disease remains one of the most important bacterial diseases affecting the poultry industry, particularly in young chicks, where infection is associated with high mortality, impaired growth performance, and substantial economic losses. Although considerable progress has been made in controlling *S. pullorum* through biosecurity measures, antibiotic therapy, vaccination, and other intervention strategies, each approach has inherent limitations that restrict its long-term effectiveness. Antibiotics remain the primary therapeutic option; however, their extensive use has contributed to the emergence of antimicrobial resistance and concerns regarding drug residues in poultry products [31]. Vaccination has also been considered an effective preventive strategy, but the protective efficacy of currently available vaccines may vary under different field conditions and immunization programs [2]. Likewise, bacteriophage therapy has emerged as a promising alternative antibacterial strategy, although its practical application is often constrained by narrow host specificity and the potential development of phage-resistant bacterial strains [32]. In contrast, probiotics have attracted considerable attention as an antibiotic alternative because they can improve intestinal health by modulating the gut microbiota, strengthening epithelial barrier function, and regulating host immune responses [33,34]. Furthermore, the use of engineered probiotics has emerged as a promising strategy for the localized delivery of therapeutic molecules within the gastrointestinal tract. In the present study, we engineered *B. subtilis* to serve not only as a probiotic carrier but also as an in situ delivery system for the host-derived mucosal repair factors gTFF2 and gEGF. This strategy retains the intrinsic probiotic properties of *B. subtilis* while providing continuous local delivery of bioactive proteins within the intestinal tract, thereby simultaneously promoting intestinal barrier repair and enhancing host resilience against *S. pullorum* infection. Therefore, engineered probiotic-mediated delivery of mucosal repair factors may represent a promising complementary preventive strategy for controlling pullorum disease in poultry production.

TFF2 and EGF are important mucosal repair factors with well-documented roles in epithelial restitution, intestinal development, and immune regulation [35,36,37,38]. However, the practical application of these bioactive factors has been limited by the low efficiency and high cost of conventional production and delivery approaches [39,40]. As a food-grade probiotic with strong gastrointestinal adaptability and protein secretion capacity, B. subtilis represents an attractive delivery platform for recombinant bioactive molecules [8,41,42]. Previous studies have demonstrated that B. subtilis supplementation can improve growth performance, intestinal health, and resistance to enteric pathogens in livestock and poultry [43,44]. Building upon these advantages, the present study developed an engineered probiotic strategy for the delivery of mucosal repair factors and demonstrated its protective efficacy against pullorum disease in young chicks.

The pHT43-based secretion system enabled extracellular production of gTFF2 and gEGF in B. subtilis, which may facilitate practical probiotic-mediated delivery of bioactive molecules without extensive purification procedures. Consistent with previous studies [45,46], protein expression was influenced by induction conditions, and optimal secretion of both recombinant proteins was achieved under 1 mM IPTG induction at 37 °C. These findings support the feasibility of using engineered B. subtilis as a stable and scalable platform for oral delivery of mucosal repair factors in poultry.

The in vitro findings suggest that gTFF2 and gEGF contribute to intestinal epithelial repair through distinct but potentially complementary biological activities. Recombinant gEGF significantly promoted DF-1 cell proliferation, consistent with the established role of EGF in supporting epithelial growth and intestinal development [47,48,49]. In contrast, gTFF2 primarily enhanced epithelial cell migration without markedly affecting proliferation, which is consistent with the classical concept of epithelial restitution in which TFF peptides facilitate rapid mucosal repair through the migration of surviving epithelial cells [50,51].

The protective efficacy of this engineered probiotic strategy was demonstrated in the chick challenge model. Previous studies have shown that prophylactic administration of probiotics can provide superior protection against enteric pathogen colonization compared with post-infection intervention [29,52]. Consistent with this concept, oral administration of recombinant B. subtilis prior to S. pullorum challenge significantly reduced mortality and suppressed cecal colonization by 2–3 orders of magnitude. These findings suggest that early-life delivery of gTFF2 and gEGF may enhance host defense against pullorum disease, potentially through improving mucosal protection and reducing intestinal damage during infection.

Excessive inflammatory responses contribute substantially to tissue injury during *S. pullorum* infection in young chicks. In the present study, oral administration of recombinant B. subtilis was associated with increased spleen and thymus indices, suggesting a potential improvement in immune development. In addition, treatment with gTFF2- and gEGF-expressing strains reduced serum IL-6 levels while increasing TGF-β levels, indicating modulation of infection-associated inflammatory responses. IL-6 is an important pro-inflammatory cytokine involved in intestinal inflammatory injury during bacterial infection [53,54], whereas TGF-β plays a critical role in limiting excessive inflammation and maintaining tissue repair processes [55]. Therefore, the reduced inflammatory response observed in the treated groups may contribute to the alleviation of tissue damage and improved host protection against pullorum disease.

Previous studies have shown that combined administration of EGF and TFF family peptides can enhance tissue repair and mucosal protection in gastrointestinal and respiratory injury models [56]. The superior protective effect observed in the gTFF2+gEGF group in the present study may likewise be attributed to the complementary biological functions of these two host-derived factors during intestinal mucosal repair. EGF primarily promotes epithelial cell proliferation through activation of the epidermal growth factor receptor (EGFR) and its downstream PI3K/Akt and MAPK/ERK signaling pathways, thereby accelerating epithelial regeneration following intestinal injury [57]. In contrast, TFF2 plays a pivotal role in epithelial restitution by stimulating the rapid migration of surviving epithelial cells to cover denuded mucosal surfaces, while also contributing to mucus barrier maintenance and mucosal integrity [58,59]. Therefore, the combination of gEGF and gTFF2 may simultaneously enhance epithelial proliferation and epithelial restitution, resulting in more efficient restoration of intestinal barrier function and improved resistance to *S. pullorum* infection. This interpretation is also consistent with our in vitro findings that gEGF predominantly promoted DF-1 cell proliferation, whereas gTFF2 exerted a stronger effect on cell migration. Moreover, chicks receiving the combined administration of gTFF2- and gEGF-expressing strains exhibited improved histopathological outcomes following *S. pullorum* infection, including reduced hepatic and intestinal lesions and better preservation of intestinal villus architecture. In particular, the combined treatment group showed increased villus height, reduced crypt depth, and an improved villus-to-crypt ratio compared with the control groups, further supporting the notion that simultaneous enhancement of epithelial proliferation and restitution contributes to protection against infection-associated intestinal injury.

Although the present study demonstrated the prophylactic efficacy of recombinant *B. subtilis*, several important aspects regarding its persistence and biosafety warrant further investigation. Previous studies have shown that *Bacillus* probiotics generally exhibit transient colonization of the gastrointestinal tract and are gradually eliminated after administration is discontinued, suggesting a relatively low risk of long-term persistence in the host [60,61]. Nevertheless, the environmental fate of recombinant strains, horizontal dissemination, and the stability of heterologous protein expression during gastrointestinal passage remain important biosafety considerations and should be systematically investigated before practical application in poultry production [62].

In conclusion, the present study demonstrates that engineered *B. subtilis* strains expressing gTFF2 and gEGF provide protective effects against *S. pullorum* infection in chicks, including reduced mortality, decreased intestinal colonization, alleviated inflammatory injury, and improved intestinal morphology. The combined administration of gTFF2- and gEGF-expressing strains showed enhanced protective efficacy against infection-associated intestinal damage. Although the present study focused on the early protective effects during the acute stage of infection, longer-term studies are warranted to evaluate the durability of protection and the long-term effects on intestinal recovery and bacterial clearance. Collectively, these findings support the potential application of engineered probiotic-mediated delivery of mucosal repair factors as a promising antibiotic-alternative preventive strategy for pullorum disease in poultry production.

## Figures and Tables

**Table 1:** Strains, plasmids, and primers used in this study.

Strain, Plasmid and Primer	Description	Source
**Strain**		
*B. subtilis* WB800N	nprE aprE epr bpr mpr::ble nprB::bsr Δvpr wprA::hyg cm::neo; NeoR	MiaoLingBio, Wuhan, China (Product No.: T0105)
pHT43-gTFF2/WB800N	*B. sbutilis* WB800N with plasmid pHT43-gTFF2	This study
pHT43-gEGF/WB800N	*B. sbutilis* WB800N with plasmid pHT43-gEGF	This study
*E. coli* DH5α	For subcloning and plasmid amplification	Preserved in our laboratory
*Salmonella pullorum* CVCC528	For the construction of animal infection models	Preserved in our laboratory
**Vector**		
pHT43-His	Cm for *B. subtilis*, Amp for *E. coli*, Pgrac01 promoter, modified signal peptide of α-amylase, with a 6×His tag.	MiaoLingBio, China (Product No.: P0704)
pHT43-gTFF2	pHT43-His+gTFF2 peptide cds (BamH I and Sma I site)	This study
pHT43-gEGF	pHT43-His+gEGF peptide cds (BamH I and Sma I site)	This study
**Primer**		
gTFF2-F	CGGGATCCATGGATCTGAAGGGGATCTGC	
gTFF2-R	TCCCCCGGGTCCCAGTTGAGCAGGTTCTTC	
gEGF-F	CGGGATCCATGGGTGACTCCATAGGATGC	
gEGF-R	TCCCCCGGGCTTCACTTACCGCTCAGCGTG	
pHT43-F	ATTCAAAAACGAAAGCGGAC	
pHT43-R	CCATTTGTTCCAGGTAAGGTAT	
InvA-F	AGGGCCTGGACGATAACAGCAT	
InvA-R	TAGCCAAGCTCCCGGAGTTTCT	

**Table 2:** Ingredients composition of the basal diet (%, as fed).

Item %	Amount %
Ingredients	
Corn	51.38
Soybean oil	3.75
Soybean meal	40.71
CaHPO_3_·2H_2_O	1.86
Limestone	1.24
NaCl	0.35
DL-Met	0.20
Vitamine premix ^a^	0.03
Trace mineral premix ^b^	0.20
50% Choline chloride	0.25
Antioxidant	0.03
Total	100
Nutrient level ^c^	
ME, MJ/kg	12.31
Crude protein	22.00
Lys	1.21
Met	0.52
Ca	1.00
Available phosphorus	0.45

^a^ Vitamin premix (1 kg) contained: vitamin A, 50 MIU; vitamin D_3_, 12 MIU; vitamin K_3_, 10 g; vitamin B_1_, 10 g; vitamin B_2_, 32 g; vitamin B_12_, 0.1 g; vitamin E, 0.2 MIU; biotin, 0.5 g; folic acid, 5 g; pantothenic acid, 50 g; niacin, 150 g. ^b^ Trace mineral premix (1 kg) contained: copper, 4 g; zinc, 90 g; iron, 38 g; manganese, 46.48 g; selenium, 0.1 g; iodine, 0.16 g; cobalt, 0.25 g. ^c^ Calculated value based on the analyzed data for the experimental diets.

**Table 3:** Gross pathological lesion scoring criteria.

Score	Gross Pathological Findings	
0	No visible gross lesions. Liver, spleen, and intestine appear normal.	
1	Mild lesions, including slight hepatic congestion or enlargement, mild intestinal hyperemia, or slight splenomegaly.	
2	Moderate lesions with obvious hepatic congestion and enlargement, focal intestinal hemorrhage, mild intestinal distension, or moderate splenomegaly.	
3	Severe lesions characterized by extensive hepatic congestion and focal necrosis, marked intestinal hemorrhage or edema, obvious intestinal distension, and pronounced splenomegaly.	
4	Very severe lesions with diffuse hepatic necrosis, extensive intestinal hemorrhage and necrosis, severe organ enlargement, or multiple severe gross lesions.	
**Score**	**Liver**	**Ileum**
0	Normal hepatic architecture; hepatocytes arranged regularly; no obvious degeneration, necrosis, congestion, or inflammatory cell infiltration.	Normal intestinal morphology; intact villi and crypts; orderly epithelial structure; no obvious inflammatory infiltration.
1	Mild lesion; scattered hepatocellular degeneration or mild cytoplasmic vacuolation; minimal inflammatory cell infiltration; hepatic architecture largely preserved.	Mild lesion; slight villus shortening or epithelial loosening; minimal epithelial shedding; mild inflammatory cell infiltration in the lamina propria.
2	Moderate lesion; evident hepatocellular degeneration and vacuolation; mild focal necrosis or congestion; moderate inflammatory cell infiltration.	Moderate lesion; partial villus disruption or epithelial shedding; mild-to-moderate crypt damage; moderate inflammatory cell infiltration.
3	Severe lesion; extensive hepatocellular degeneration, obvious focal necrosis, marked congestion/hemorrhage, prominent inflammatory infiltration; hepatic cords partially disrupted.	Severe lesion; obvious villus rupture or fusion, extensive epithelial shedding, marked crypt damage, and prominent inflammatory infiltration in the lamina propria.
4	Very severe lesion; diffuse hepatocellular necrosis, extensive inflammatory infiltration, severe hemorrhage/congestion, and marked disruption of hepatic architecture.	Very severe lesion; extensive villus destruction or loss, severe epithelial necrosis/shedding, crypt collapse, mucosal erosion, and diffuse inflammatory cell infiltration.

**Table 4:** The BW (g) and ADG (g) in different treatment groups of chicks.

Items	Blank Control	PBS	pHT43	gTFF2	gEGF	gTFF2+gEGF
BW on day 0	37.3292 ± 4.20 a	37.72 ± 4.48 a	38.45 ± 3.91 a	37.23 ± 4.15 a	36.63 ± 7.37 a	36.09 ± 3.15 a
BW on day 5	55.83 ± 5.45 ab	54.07 ± 6.08 a	58.09 ± 5.62 abc	58.89 ± 4.30 bc	61.20 ± 7.29 c	61.97 ± 3.03 c
ADG on day 0–5	3.45 ± 0.63 a	3.27 ± 1.08 a	3.93 ± 1.56 ab	4.33 ± 1.43 ab	4.91 ± 1.16 b	5.18 ± 0.74 b

Note: ^a,b,c^ Different superscripts within a row indicate significantly different means (*p* < 0.05). The results are reported as the mean ± SD for 12 chicks per group.

**Table 5:** Immune organ indices in different treatment groups of chicks.

Index (g/kg)	Blank Control	PBS	pHT43	gTFF2	gEGF	gTFF2+gEGF
Spleen index	2.817 ± 0.061 ^ab^	2.416 ± 0.213 ^a^	2.379 ± 0.095 ^a^	3.275 ± 0.293 ^b^	3.467 ± 0.162 ^bc^	4.139 ± 0.123 ^c^
Thymus index	2.793 ± 0.116 ^abc^	2.379 ± 0.216 ^a^	2.759 ± 0.223 ^ab^	3.149 ± 0.069 ^bcd^	3.872 ± 0.031 ^d^	3.437 ± 0.073 ^cd^
Bursa of Fabricius index	3.155 ± 0.211	2.855 ± 0.213	2.918 ± 0.051	3.276 ± 0.137	3.359 ± 0.227	3.568 ± 0.218

Note: ^a,b,c,d^ Different superscripts within a row indicate significantly different means (*p* < 0.05). The results are reported as the mean ± SD for 3 chicks per group.

**Table 6:** Mean villous height and crypt depth in different compartments of the ileum after the treatments.

Items	Blank Control	PBS	pHT43	gTFF2	gEGF	gTFF2+gEGF
villous height	367.6 ± 24.76 ^b^	228.4 ± 23.18 ^a^	235.2 ± 33.80 ^a^	457.1 ± 38.12 ^c^	499.1 ± 31.02 ^c^	590.4 ± 37.61 ^d^
crypt depth	81.92 ± 7.621 ^b^	72.38 ± 9.236 ^ab^	99.04 ± 12.77 ^b^	56.92 ± 9.692 ^ab^	57.14 ± 9.212 ^a^	55.23 ± 3.807 ^a^
villous height/ crypt depth	4.289 ± 0.951 ^ab^	3.244 ± 0.577 ^a^	2.514 ± 1.057 ^a^	8.028 ± 0.985 ^b^	8.944 ± 1.556 ^c^	10.68 ± 0.315 ^c^

Note: ^a,b,c^ different superscripts within a row indicate significantly different means (*p* < 0.05). The results are reported as the mean ± SD for 3 chicks per group.

## Data Availability

The raw data supporting the conclusions of this article will be made available by the authors on request.

## Supplementary Materials

The following supporting information can be downloaded at: https://www.mdpi.com/article/10.3390/microorganisms14081606/s1, Figure S1. Gel electrophoresis analysis of gTFF2 (384 bp) and gEGF (516 bp) gene PCR products. (a) Lane M, DL 5000 marker. Lane 1–11, amplification products of gTFF2. Lane 12, negative control. (b) Lane M, DL 2000 marker. Lane 1–5, amplification products of gEGF. Lane 6, negative control. Figure S2. PCR identification of recombinant plasmids. (a) Gel electrophoresis analysis of pHT43-gTFF2 plasmid PCR products. Lane M, DL 2000 marker. Lane 1-5, amplification products of pht43-gTFF2 plasmid. Lane 6, negative control. (b) Agarose gel electrophoresis of pHT43-gTFF2 plasmid by double enzyme digestion. Lane M, DL 8000 marker. Lane 1, pHT43-gTFF2 plasmid digested by restriction enzymes BamH I and Sma I. (c) Gel electrophoresis analysis of pHT43-gEGF plasmid PCR products. Lane M, DL 2000 marker. Lane 1–6, amplification products of pHT43-gEGF plasmid. Lane 7, negative control. (d) Agarose gel electrophoresis of pHT43-gEGF plasmid by double enzyme digestion. Lane M, DL 8000 marker. Lane 1, pHT43-gEGF plasmid digested by restriction enzymes BamH I and Sma I.
